# The effects of nitrogen and potassium nutrition on the growth of nonembryogenic and embryogenic tissue of sweet orange (*Citrus sinensis *(L.) Osbeck)

**DOI:** 10.1186/1471-2229-8-126

**Published:** 2008-12-16

**Authors:** Randall P Niedz, Terence J Evens

**Affiliations:** 1 United States Department of Agriculture, Agricultural Research Service, US Horticultural Research Laboratory, Ft. Pierce, FL 34945-3030, USA

## Abstract

**Background:**

Mineral nutrients are one of the most basic components of plant tissue culture media. Nitrogen in the form of NH_4_^+ ^and NO_3_^- ^is the dominant mineral nutrient in most plant tissue culture formulations, with effects dependent on both the proportion and the amount of NH_4_^+ ^and NO_3_^-^. The effects of nitrogen nutrition on the growth of nonembryogenic and embryogenic cell lines of sweet orange (*C. sinensis *(L.) Osbeck cv. 'Valencia'), tissues routinely used in citrus horticultural and plant improvement research, was explored using an experimental approach free of ion confounding that included a 2-component mixture (NH_4_^+^:K^+^) and a quantitative factor [NO_3_^-^] crossed by the mixture, thereby providing ion-specific estimates of proportional and amount effects.

**Results:**

First, the linear mixture component, though only a comparison of the design space vertices, was highly significant for both tissue types and showed that NH_4_^+ ^was required by both tissues. Second, the NH_4_^+ ^* K^+ ^mixture term was highly significant for both tissue types, revealing that NH_4_^+ ^and K^+ ^exhibit strong synergistic blending and showed that growth was substantially greater at certain blends of these two ions. Third, though the interaction between the NH_4_^+^:K^+ ^mixture and NO_3_^- ^amount on fresh weight accumulation for both tissue types was significant, it was substantially less than the main effect of the NH_4_^+^:K^+ ^mixture. Fourth, a region of the design space was identified where fresh weight growth was increased 198% and 67% over the MS medium controls for nonembryogenic and embryogenic tissues.

**Conclusion:**

By designing a mineral nutrient experiment free of ion confounding, a direct estimation of ion-specific proportional and amount effects on plant tissue growth is possible. When the ions themselves are the independent factors and/or mixture components, the resulting design space can be systematically explored to identify regions where the response(s) is substantially improved over current media formulations. In addition, because the response is over a defined experimental region, a specific medium formulation is more accurately interpreted as a coordinate in the specified design geometry.

## Background

Mineral nutrients are one of the most basic components of plant tissue culture media. Unlike carbon sources, plant growth regulators, vitamins, amino acids, gelling agents and undefined substances that may or may not be included in any given medium, the mineral nutrients are always present [1]. Thus, a great deal of time and effort has been devoted to identifying the optimal concentrations for each of the currently established 14 essential plant nutrients [2]. Nitrogen in the form of NH_4_^+ ^and NO_3_^- ^is the dominant mineral nutrient in most tissue culture formulations including MS [3] the most widely used nutrient formulation in plant tissue culture. Nitrogen effects are highly dependent on both the total amount of nitrogen and on the proportion of NH_4_^+ ^and NO_3_^- ^and affect a wide range of in vitro responses including callus growth, shoot and root organogenesis, embryogenesis, and shoot multiplication [1]. We thus chose to determine the effects of nitrogen nutrition on the growth of nonembryogenic and embryogenic cell lines of sweet orange (*C. sinensis *(L.) Osbeck).

Nonembryogenic tissue has been used for biochemical characterization of pathogenesis-related (PR) proteins [4] and as a source of protoplasts for somatic hybridization [5]. Embryogenic tissue is often used for enzymatic studies [6,7] is the primary source of protoplasts for somatic hybridization [8] and is also used for genetic transformation [9].

A primary consideration in quantifying the effects of specific mineral nutrients is the concept of ion confounding as previously discussed in [10] and [11]. Ion confounding occurs when salts are treated as experimental factors in experimental designs focused on determining the effects of nutrients/ions in solution. To illustrate this concept, consider a simple experiment wherein a single salt such as KNO_3 _is varied over some concentration range and a particular *in vitro *response is measured. Any measured change in the response may be due to K^+^, NO_3_^-^, and/or the interaction between K^+ ^*and *NO_3_^-^. When salts are used as factors both ions are simultaneously varied; consequently, their effects are potentially confounded with each other [12,13]. No valid conclusions can be derived regarding the main effects of the two component ions K^+ ^or NO_3_^- ^or their interaction from such an experiment. The measured effect in a salt-based experiment is actually the mean effect of the two ions, K^+ ^and NO_3_^-^, in a 1:1 proportion at varying concentrations. The so-called "co-ion approach" often employed to circumvent this limitation is not valid [11]. In short, ion confounding occurs when the ion(s) of interest are covaried with the complementary ion(s) associated with the salts used; that is, attempting to vary the concentration of a single cation or anion using a salt results in a simultaneous change in the associated co-ion. Such changes also include ions added via pH adjustments but unaccounted for in the experimental design. We report the results from an approach that, to the best of our knowledge, is the first study on the effects of nitrogen nutrition obtained with experimentation free of ion confounding.

## Results

### Nonembryogenic callus

The percentage increase of the fresh weight of nonembryogenic (NE) sweet orange callus over fourteen days ranged from 2% – 926% (Table 1), indicating that K^+^, NH_4_^+^, and NO_3_^- ^nutrition are important regulators of NE callus fresh weight growth. For % fresh weight increase the best fitting polynomial was a reduced quadratic mixture × cubic process response surface obtained by backward elimination. A summary of the ANOVA, lack-of-fit test and three R^2 ^statistics for % fresh weight increase and dry weight are presented in Table 2. A single point (Table 1: #14) was identified as suspect by the outlier-t test [14] and was ignored in the fresh and dry weight analyses.

**Table 1:** Mixture-amount treatment points and fresh- and dry-weight data for NE (nonembryogenic callus) and E (embryogenic callus).

**Treatment Design Points**	**Block**	**Mixture Components**		**Factor**	**Fresh Wgt (Increase %)**		**Dry Wgt (g)**		**pH**^a^
			
		**NH_4_^+^**	**K^+^**	**NO_3_^+ ^mM**	**NE**	**E**	**NE**	**E**	
1	1	0.250	0.750	10.00	387	288	0.141	0.183	5.7
2	1	0.500	0.500	50.00	329	344	0.125	0.243	5.8
3	1	0.500	0.500	30.00	317	539	0.127	0.176	5.8
4	1	0.000	1.000	20.00	13	23	0.066	0.074	5.7
5	1	0.500	0.500	10.00	408	315	0.140	0.146	5.7
6	1	0.250	0.750	30.00	926	520	0.266	0.262	5.8
7	1	0.250	0.750	30.00	885	490	0.246	0.256	5.8
8	1	0.250	0.750	50.00	425	313	0.152	0.252	5.8
9	1	0.500	0.500	30.00	406	391	0.143	0.154	5.8
10	1	0.000	1.000	50.00	15	33	0.059	0.062	5.8
11	1	0.250	0.750	10.00	360	332	0.160	0.208	5.7
12	1	0.500	0.500	50.00	290	333	0.122	0.244	5.8
13	1	0.000	1.000	50.00	13	33	0.066	0.061	5.8
14	2	0.500	0.500	10.00	406	52	0.149	0.068	5.7
15	2	0.000	1.000	10.00	10	34	0.058	0.092	5.7
16	2	0.125	0.875	30.00	191	295	0.106	0.245	5.8
17	2	0.500	0.500	40.00	99	298	0.073	0.245	5.8
18	2	0.250	0.750	50.00	115	280	0.080	0.234	5.8
19	2	0.250	0.750	50.00	205	282	0.110	0.242	5.8
20	2	0.500	0.500	40.00	123	308	0.079	0.259	5.8
21	2	0.500	0.500	10.00	208	202	0.107	0.12	5.7
22	2	0.000	1.000	40.00	2	23	0.044	0.071	5.8
23	2	0.000	1.000	10.00	12	24	0.056	0.084	5.7
24	2	0.000	1.000	10.00	9	33	0.050	0.096	5.7
25	2	0.250	0.750	20.00	403	493	0.156	0.241	5.8
26	2	0.000	1.000	40.00	15	71	0.0702	0.095	5.8

Experiment is a two-component mixture of NH4+ and K+ and one quantitative factor NO3- amount. The mixture components are listed as proportions with their actual amounts matched to the amount of NO3-. For example, treatment point #1 included 2.5 mM NH4+, 7.5 mM K+, and 10 mM NO3-. The data represent the mean of six duplicate plates per treatment point. Points #17 and #20 are MS medium.

^a ^– Calculated from the chemical equilibrium modeling software MINEQL+ Ver. 4.5 (27), temperature corrected and assumed open to the atmosphere with a P_CO2 _at sea level of 10^-3.50 ^atm.

**Table 2:** ANOVA, regression coefficients, and summary statistics for percentage fresh weight increase and dry weights of nonembryogenic tissue.

**Source**	**Nonembryogenic tissue**					
	
	**Fresh Wgt.^a^**			**Dry Wgt.^a^**		
	
	**F Value**	**p-values**	**Regression coefficients^c^**	**F Value**	**p-values**	**Regression coefficients^c^**
Model	87.23	< 0.0001		22.18	< 0.0001	
Linear Mixture	279.34	< 0.0001		50.77	< 0.0001	
NH_4_			+ 15.24			- 0.98
K			+ 2.63			- 1.20
NH_4 _* K	290.91	< 0.0001	+ 70.70	64.35	< 0.0001	+ 1.60
NH_4 _* [NO_3_]	7.72	0.0148	- 1.72	2.53	0.1326	- 0.049
K * [NO_3_]	15.50	0.0015	+ 8.34	1.49	0.2409	- 0.035
NH_4 _* K * [NO_3_]	10.37	0.0062	+ 9.99	0.54	0.4754	+ 0.11
NH_4 _* [NO_3_]^2^	0.16	0.6979	+ 0.41	0.20	0.6593	+ 0.023
K * [NO_3_]^2^	0.53	0.4771	+ 0.93	0.29	0.5950	- 0.034
NH_4 _* K * [NO_3_]^2^	70.53	< 0.0001	- 44.15	12.10	0.0034	- 0.89
K * [NO_3_]^3^	24.16	0.0002	- 11.45	-	-	

Lack of Fit	p = 0.2641			p = 0.3634		
R^2^	0.98			0.92		
R^2 ^adj	0.97			0.88		
R^2 ^pred	0.95			0.78		
Std. Dev.	1.28			0.06		
Mean	13.32			-1		
C.V. %	9.63			6.33		
Model type	reduced quadratic × cubic^b^			quadratic × quadratic		

^a ^Data transformation was determined by a Box Cox plot analysis – fresh weight data were transformed by square root and NE dry weight by log base 10.

^b ^Model reduction by backward elimination.

^c ^Presented in coded form. Coding normalizes the factor ranges by placing their low and high range value between -1 and +1 and can thus, be directly compared.

**Figure 1:**
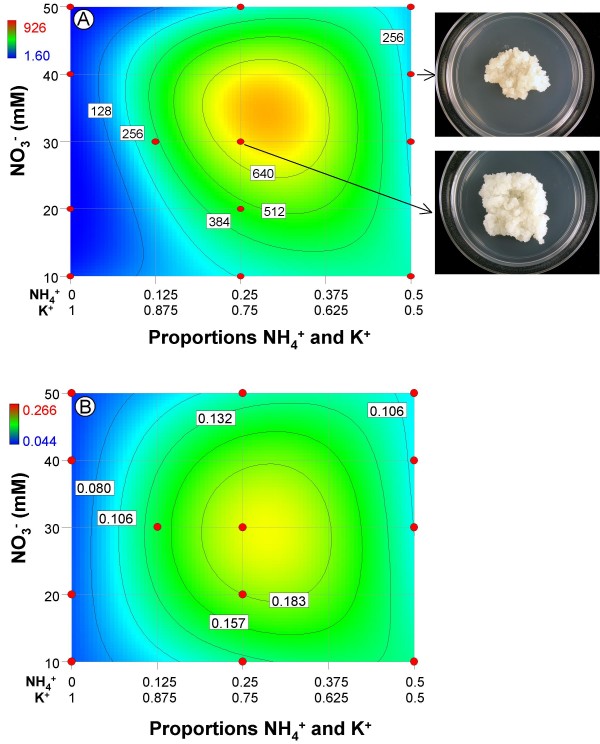
**Fresh and dry weight response contour plots for nonembryogenic tissue**. A) % increase in fresh weight growth; B) dry weight. Pictures of the difference in biomass between standard MS and the center point of the experimental design space are pictured to the right of each plot.

Fresh weight growth (Fig. 1A) required a square root transformation as per a Box-Cox power transform plot analysis. The residual and model diagnostics were within acceptable limits [15]. The lack-of-fit test was not significant (p = 0.2641) indicating that additional variation in the residuals could not be removed with a better model. R^2^, R^2^_adj _and R^2^_pred _statistics ranged from 0.95 – 0.98. The overall model was highly significant (p < 0.0001), indicating NH_4_^+^, K^+^, and NO_3_^- ^significantly affected growth. The ANOVA contained seven significant terms; three of the terms, the linear mixture, NH_4 _* K, and NH_4 _* K * [NO_3_]^2^, had highly significant p-values (i.e. < 0.0001; Table 2).

**Figure 2:**
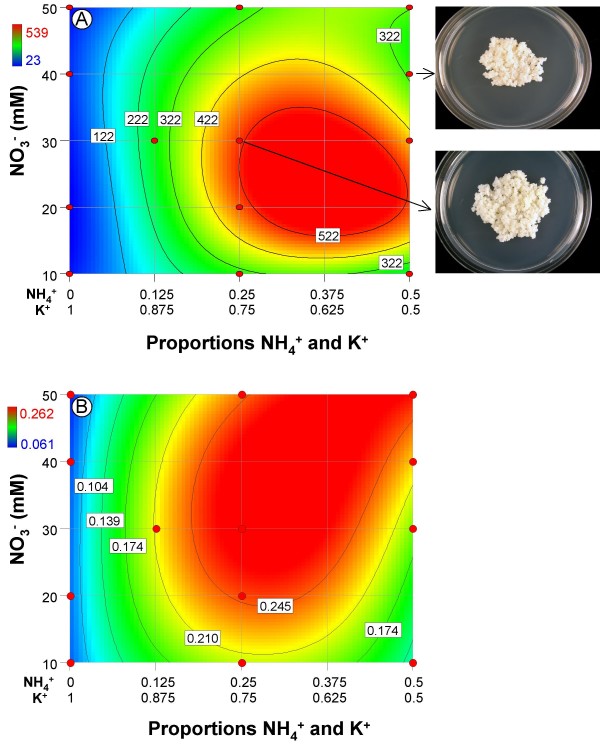
**Fresh and dry weight response contour plots for embryogenic tissue**. A) % increase in fresh weight growth; B) dry weight. The standard MS point and the point of greatest growth are indicated on each plot.

Dry weight accumulation (Fig. 2A) ranged from 0.04 g – 0.27 g and required a log base 10 transformation as per a Box-Cox power transform plot analysis. Model diagnostics were within acceptable limits and the lack-of-fit test was not significant (p = 0.3634), indicating that additional variation in the residuals could not be removed with a better model. R^2^, R^2^_adj _and R^2^_pred _statistics ranged from 0.78 – 0.92, indicating good agreement between these three values. The overall model was highly significant (p < 0.0001) indicating significant factor effects on dry weight by these three ions. The ANOVA revealed three significant terms; two of which, the linear mixture and NH_4 _* K terms, had p-values < 0.0001 (Table 2).

### Embryogenic callus

The percentage increase of the fresh weight of embryogenic (E) sweet orange callus over fourteen days ranged from 23% – 539% (Table 1), indicating that K^+^, NH_4_^+^, and NO_3_^- ^nutrition are important regulators of this response. For % fresh weight increase, the best fitting polynomial was a reduced quadratic × cubic (mixture × numeric factor) response surface obtained by backward elimination. A summary of the ANOVA, lack-of-fit test and three R^2 ^statistics are presented in Table 3. Fresh weight growth data required a square root transformation as per a Box-Cox power transform plot analysis. Model diagnostics were within acceptable limits and the lack-of-fit test was not significant (p = 0.3024) indicating that additional variation in the residuals could not be removed with a better model. The three R^2 ^statistics ranged from 0.8 – 0.98. The overall model was highly significant (p < 0.0001), indicating NH_4_^+^, K^+^, and NO_3_^- ^significantly affected growth. The ANOVA revealed six significant terms; two of which, the linear mixture and NH_4 _* K had highly significant p-values (Table 3). Fresh weight growth over the design space is shown in Fig. 1B.

**Table 3:** ANOVA, Regression coefficients, and summary statistics for percentage fresh weight increase and dry weights of embryogenic tissue.

**Source**	**Embryogenic tissue**					
	
	**Fresh Wgt.^a^**			**Dry Wgt.^a^**		
	
	**F Value**	**p-values**	**Regression coefficients^c^**	**F Value**	**p-values**	**Regression coefficients^c^**
Model	59.82	< 0.0001		49.07	< 0.0001	
Linear Mixture	350.72	< 0.0001		153.04	< 0.0001	
NH_4_			+ 21.15			+ 0.19
K			+ 6.01			+ 0.085
NH_4 _* K	73.69	< 0.0001	+ 36.18	86.33	< 0.0001	+ 0.54
NH_4 _* [NO_3_]	5.68	0.0330	- 6.89	53.20	< 0.0001	+ 0.065
K * [NO_3_]	2.38	0.1471	+ 3.15	0.65	0.4339	- 6.69E-03
NH_4 _* K * [NO_3_]	0.42	0.5276	- 1.97	0.76	0.3964	- 0.038
NH_4 _* [NO_3_]^2^	15.18	0.0018	- 4.13	0.24	0.6279	+ 7.27E-03
K * [NO_3_]^2^	0.11	0.7409	- 0.42	0.27	0.6102	- 9.41E-03
NH_4 _* K * [NO_3_]^2^	5.76	0.0321	- 12.44	6.93	0.0188	- 0.20
NH_4 _* [NO_3_]^3^	6.95	0.0205	+ 7.95	-	-	-
K * [NO_3_]^3^	2.23	0.1596	- 3.35	-	-	-

Lack of Fit	p = 0.3024			p = 0.0036		
R^2^	0.98			0.96		
R^2 ^adj	0.96			0.94		
R^2 ^pred	0.80			0.89		
Std. Dev.	1.24			0.018		
Mean	14.53			0.17		
C.V. %	8.52			10.59		
Model type	reduced quadratic × cubic^b^			quadratic × quadratic		

^a ^Data transformation was determined by a Box Cox plot analysis – fresh weight data were transformed by square root and NE dry weight by log base 10.

^b ^Model reduction by backward elimination.

^c ^Presented in coded form. Coding normalizes the factor ranges by placing their low and high range value between -1 and +1 and can thus be directly compared.

Dry weight accumulation (Fig. 2B) required a log base 10 transformation as per a Box-Cox power transform plot analysis. Model diagnostics were within acceptable limits and the lack-of-fit test was significant (p = 0.0036) indicating that 1) additional variation in the residuals might be accounted for with a better model or, 2) an unusually low level of pure error was present. The three R^2 ^statistics ranged from 0.89 – 0.96. The overall model was highly significant (p < 0.0001) indicating significant factor effects on dry weight accumulation by these three ions. The ANOVA revealed four significant terms, three of which, the linear mixture, NH_4 _* K, and NH_4 _* [NO_3_^-^], had p-values < 0.0001 (Table 3).

### Analysis

The effects of these three ions on nonembryogenic and embryogenic tissue growth were similar in several ways. First, the linear mixture component was highly significant for both tissue types. The linear mixture component compares the responses at the extreme ends (vertices) of the mixture design space. This means that growth at the points comprising the 0 NH_4_^+^:1 K^+ ^ratio were compared to growth at the points comprising the 0.5 NH_4_^+^:0.5 K^+ ^ratio. Likewise, the regression coefficients for NH_4_^+ ^and K^+ ^in Tables 2 and 3 are estimates of growth at the vertices only, not estimates of the effects of these components. It is clear when viewing Figure 1 that growth along the 0 NH_4_^+^:1 K^+ ^y-axis is considerably less than growth along the 0.5 NH_4_^+^:0.5 K^+ ^y-axis; this is reflected in the larger regression coefficient for NH_4_^+ ^vs. K^+ ^in Tables 2 and 3. Good growth of citrus nonembryogenic and embryogenic tissue requires that [NH_4_^+^]>0, which is consistent with many other tissue culture systems [1].

Second, the NH_4_^+ ^*K^+ ^term was highly significant for both tissue types (Tables 2, 3), which reveals that NH_4_^+ ^and K^+ ^exhibit strong synergistic blending. This means that growth was substantially greater at certain blends of these two ions vs. the growth that was observed at the extreme ends or vertices of the mixture. For the two cell lines, ratios from 0.250 NH_4_^+^:0.750 K^+ ^to 0.375 NH_4_^+^:0.625 K^+ ^resulted in the greatest increase in fresh weight (Figs. 1, 2). These ranges correspond to a NH_4_^+^:NO_3_^- ^ratio of 1:3 at 37.5 mM total N at the centerpoints (i.e., the points where the greatest growth was recorded) – note that in standard MS medium this ratio is 1:2 at 60 mM total N (Figs. 1, 2). It should be noted that the NH_4_^+^:NO_3_^- ^ratio effect is only correlative and cannot be directly quantified from our experimental design.

Third, the effect of the NH_4_^+^:K^+ ^mixture and NO_3_^- ^amount on dry weight accumulation was comparable to fresh weight accumulation of nonembryogenic callus. Specifically, the NH_4_^+^:K^+ ^mixture was the primary driver of dry weight accumulation. One difference was that NO_3_^- ^amount had less of an effect on dry weight than it did for fresh weight accumulation. This result possibly suggests that the NH_4_^+^:K^+ ^mixture promotes cell division and NO_3_^- ^amount promotes cell expansion. Resolution of this effect cannot be done with the experimental design used in this study. To do this would require that all proportion effects be accounted for in an NH_4_^+^:K^+^:NO_3_^- ^mixture-amount design, which would capture the two currently "hidden" two- and three-component effects, namely, K^+^:NO_3_^-^, NH_4_^+^:NO_3_^-^, and NH_4_^+^:K^+^:NO_3_^-^. For embryogenic callus the results were somewhat different; there was a relatively strong NH_4_^+ ^* NO_3_^- ^amount effect on dry weight not observed for fresh weight (Figure 1B vs. 2B).

Interestingly, the interaction between the NH_4_^+^:K^+ ^mixture and NO_3_^- ^amount for fresh weight growth as revealed in the NH_4_^+ ^* K^+ ^* [NO_3_^-^] and NH_4_^+ ^* K^+ ^* [NO_3_^-^]^2 ^terms was significant for the nonembryogenic tissue, but not significant for the embryogenic callus. This probably reflects the greater effect of these factors on nonembryogenic tissue where the point of greatest growth was a 198% increase in fresh weight vs. a 67% increase for embryogenic tissue. It should be pointed out that the magnitude of the effects of the interaction between NH_4_^+^:K^+ ^mixture and NO_3_^- ^amount on fresh weight accumulation for both tissue types was substantially less than the main effects of the NH_4_^+^:K^+^mixture – evident when the p-values are compared.

## Discussion

The effects of NH_4_^+^, K^+^, and NO_3_^- ^on the growth of nonembryogenic and embryogenic citrus callus were determined using an approach that removed ion confounding from the experimental design. The basic approach was to 1) design an experiment where the ions NH_4_^+^, K^+^, and NO_3_^-^, as opposed to their salts [16], were the factors to be varied [11]; 2) fix all other inorganic ions at their MS levels; 3) calculate the salt/acid/base formulations required to achieve the ion levels specified for each treatment combination using the ion/salt linear programming algorithm and the software ARS-Media [10] to remove ion confounding. Because mineral nutrients are known to include both proportional and amount effects, proportionality and amount were incorporated into the design of the experiment. Because the three ions are all monovalent, treating NH_4_^+ ^and K^+ ^as a 2-component mixture matched to the amount of NO_3_^- ^resulted in a design space of near uniform pH. The effects of total nitrogen and the ratio of NH_4_^+ ^to NO_3_^- ^were indirectly captured in the selected design. It is important to point out that this approach did not directly control pH, i.e. treat pH as an independent variable. All the bulk solution properties of the initial media solutions such as pH and ion speciation were inherent to the selected design space. Because the bulk solution properties were treated as dependent variables, the experiment was free of ion confounding and allowed estimation of ion-specific effects on tissue growth. However, it is important to note that by 'constraining' pH in this manner we were unable to directly quantify the effects of the NH_4_^+^:NO_3_^- ^ratios, i.e. we can only perform correlative analyses to explore these effects.

**Figure 3:**
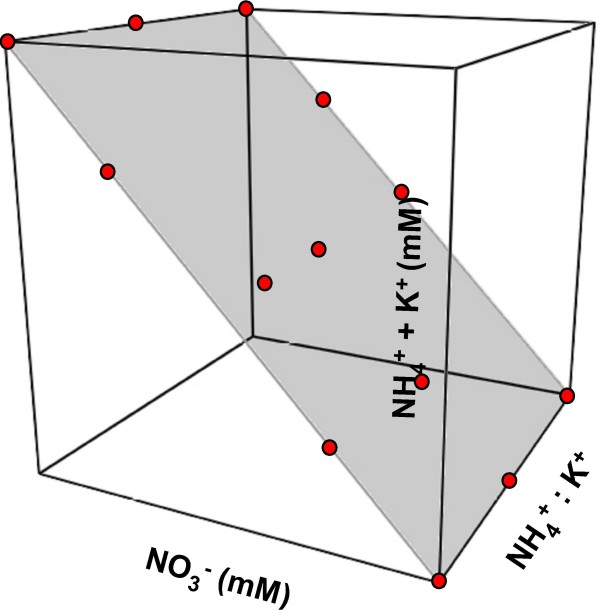
**3-dimensional design space defined by NH_4_^+^:K^+^, NH_4_^+^:K^+ ^amount, and NO_3_^- ^amount**. The experimental region for this study is a selected slice, shown in grey with treatment points in red, through the 3-dimensional design space where all points on the plane have a pH of 5.8 – pH increases above the plane and decreases below it.

We assumed when designing the experiment for this study that most of the K^+ ^ions would primarily affect bulk solution properties (as opposed to the μmolar amounts required to meet tissue nutritional requirements) such as the electrical charge of the system. The experimental design space explored in this study is actually a subset of points that describe a plane through a less-constrained 3-dimensional experimental design space defined by the axes NH_4_^+^:K^+^, NH_4_^+^:K^+ ^amount, and NO_3_^- ^amount (Fig. 3). All of the points falling on this plane have a pH near 5.8 – pH increases above the plane and decreases below it. This raises the question of the importance of the starting pH of the culture medium. Given that tissue growth was not uniform across the experimental design space, tissue growth did not correlate well with initial solution pH. The nonuniform growth across a common pH plane is analogous to the surprisingly low correlation between solution pH and protein precipitation observed in experiments free of ion confounding designed to detect ion-specific effects [11]. For the responses measured in this study, the primary drivers are the ions under independent control (i.e. NH_4_^+^, K^+ ^and NO_3_^-^) and is an empirical demonstration that pH is a dependent variable and must be treated as such experimentally. The pH, or 'relative proton activity', of a given solution is primarily determined by the type and concentration of the ions in solution. Thus, pH can only be examined in a correlational relationship and cannot be established as a causal factor. One implication of these results is that an experimental design to grow plant tissues on mineral nutrient combinations free of ion confounding greatly expands the experimental design space by removing the "pH bias" (Fig. 3). Are there regions in the experimental design space described in Figure 3 where citrus tissue would grow as well or better than on the plane sampled in these experiments? Given that each of the responses displayed a 'hot-spot' near the center of the experimental design space, there is no reason to assume that there may not be even better regions for growth that lie above or below this plane. Certainly, by sampling the full cubic design space depicted in Figure 3, regions outside of pH 5.8 where citrus tissue grows well might be identified. Thus, the selection of the "pH 5.8" plane was, to some extent arbitrary, and was chosen because it was the pH value used by Murashige and Skoog [3]. The value of the experiments conducted by Murashige and Skoog was that most of the components required for culturing a wide range of plant species in vitro were identified, and a formulation was developed that works in some fashion for a large variety of plant species. MS has been very useful as a starting point for species-specific media formulation optimization studies. The fact that the best media formulation for citrus tissue growth differs from MS is not surprising given that MS medium was developed for tobacco pith callus as opposed to citrus callus. However, because MS was developed using salt-based, pH-adjusted, one-factor-at-a-time (OFAT) experiments, we really have no reason to assume that MS is optimal even for tobacco pith callus. The research presented here is a logical extension of Murashige and Skoog's seminal work, and represents the next step in the evolution of plant tissue culture media development.

## Conclusion

A substantial increase in tissue growth was observed in sweet orange nonembryogenic and embryogenic tissue in certain regions of a 2-dimensional design space defined by 2-component NH_4_^+^:K^+ ^mixture and NO_3_^- ^amount axes. Such an approach removes ion confounding, treats all initial bulk solution properties as dependent variables, and separates proportional and amount effects. The result is an experimental design space defined by ion factors/components suited for systematic exploration. Some of the implications of this approach include 1) the practical aspects of developing improved media formulations; 2) the more basic aspects of quantifying ion-specific responses in an experimentally rigorous manner and relating these responses changes to gene/protein/metabolite profiles and phenotype and; 3) the concept that specific media formulations are more properly viewed as "ion-coordinates" in a hyperdimensional geometry defined by all of the components that constitute a medium rather than as the salt recipe that is used to create that medium.

## Methods

### Plant Material and Tissue

#### Nonembryogenic cell line

A five year old nonembryogenic cell line was developed from epicotyl explants of vitro grown seedlings of *Citrus sinensis *(L.) Osbeck cv. 'Valencia.' Seed were germinated in MS basal medium without plant growth regulators and supplemented with 3% (w/v) sucrose. One cm epicotyl explants were excised from 15–21 d old seedlings and placed onto MT medium [17] supplemented with 2.5 μM 2,4-dichlorophenoxyacetic acid (2,4-D), 1 μM 6-benzylaminopurine (BA) and 100 mg L^-1 ^casein hydrolysate. The cultures were grown in a temperature-controlled growth cabinet at 27°C on a 4-h photoperiod under low light (15–20 μmol photons m^-2 ^s^-1^) that was provided by cool-white fluorescent lamps. After 6 months of selection, rapidly growing tissue was obtained. For maintenance, the 2,4-D concentration was reduced to 1 μM.

#### Embryogenic cell line

A three year old embryogenic callus line derived from *C. sinensis *cv. 'Valencia' was initiated as described by [18]. The line was maintained on Murashige and Tucker's (MT) basal medium [17] at 27°C, in the dark, and on a 28-d subculture cycle.

To acclimate the tissue to each test formulation and minimize possible carry-over effects, experiments were initiated by first culturing approximately 1 g of callus onto each treatment formulation (or "design point"), using 100 × 15 mm polystyrene culture dishes, followed by two additional transfers. The result was that prior to experimentation the tissue used was acclimated to each treatment formulation for three fourteen day growth cycles. Following the acclimation cycles, approximately 1 g from the acclimated cultures was subcultured again onto each treatment formulation and allowed to grow for 14 days before the biomass was harvested. Fresh and dry weights were quantified by taking the average of six pseudoreplicate plates for each treatment point. Percent increase in fresh weight was calculated using the initial subcultured weight of the callus.

### Experimental Approach, Design, and Analysis

**Figure 4:**
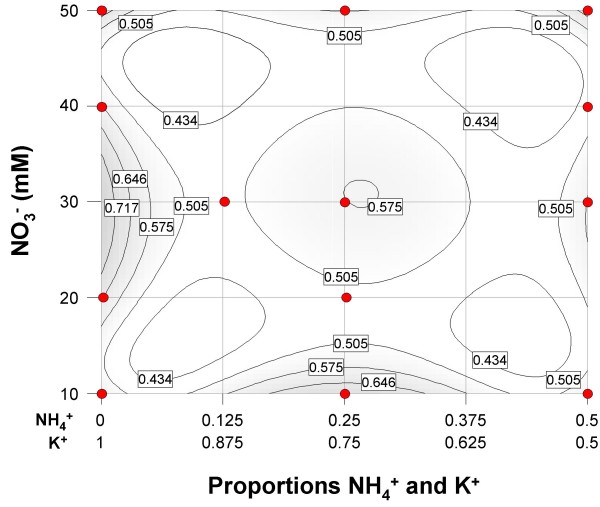
**Experimental design space with treatment points**. NH_4_^+^:K^+ ^mixture- NO_3_^- ^amount design space with contours of the standard error of prediction. The standard error of prediction showed is < 1 across the design space.

The experiment was designed as a mixture-amount [19,20] and included two mixture components, K^+ ^and NH_4_^+^, and one numeric factor, NO_3_^- ^concentration. Because K^+ ^and NH_4_^+ ^were treated as components of a mixture, the range for each component is expressed as a proportion; all component proportions in each mixture sum to one. NO_3_^- ^concentration ranged from 10 to 50 mM, K^+ ^proportion ranged from 0.5 to 1.0 and NH_4_^+ ^proportion ranged from 0.0 to 0.5. The concentration of K^+ ^plus NH_4_^+ ^was matched to the NO_3_^- ^concentration to maintain charge neutrality. No pH adjustments were required since pH was uniform across formulations. Design points were selected using D-optimal criteria to satisfy a quadratic polynomial for the mixture (NH_4_^+^:K^+^) and the numeric factor, [NO_3_^-^] crossed by the mixture; the resulting design space is depicted in Figure 4. The experiment included 8 model points, 5 lack-of-fit points, 13 points to estimate pure error, and a point for MS basal medium. The experiment included two blocks to account for the number of treatments that could be managed at one time; several treatments were repeated across the two blocks to provide estimates of block effects.

All solution recipes were derived using the linear programming approach described by [10]. The salts/acids/bases required to make each point in the design space was calculated using ARS-Media (Ver. 1.0) ion solution calculation software, which is available as a free download via , a software application specifically designed for these types of calculations [10]. For each treatment, all ions present and their amounts were entered into ARS-Media. Ions other than those being varied were fixed at their MS levels. Table 4 illustrates four examples, including MS medium, of the ion types and concentrations that were entered into ARS-Media. Preliminary tests showed that once all the organics and growth regulators were added, 3 mM Na^+ ^was required to bring the pH of the medium to 5.8. Therefore, we added 3 mM Na^+ ^to the 0.202 mM Na^+ ^already present in MS for a total of 3.202 mM. Thus, the resulting formulations calculated by ARS-Media did not require any pH adjustment as the correct amount of Na^+ ^was already incorporated into each formulation.

**Table 4:** Ion values (mM) for four treatments, including MS medium, used to solve the linear programming algorithm utilized by ARS-Media.

**Ion**	**MS**	**1**	**2**	**3**
B(OH)_3_	0.100259	0.100259	0.100259	0.100259
Ca(2+)	2.992884	2.992884	2.992884	2.992884
Cl(-)	5.985982	5.985982	5.985982	5.985982
Co(2+)	0.000105	0.000105	0.000105	0.000105
Cu(2+)	0.0001	0.0001	0.0001	0.0001
EDTA	0.100027	0.100027	0.100027	0.100027
Fe(2+)	0.099997	0.099997	0.099997	0.099997
I(-)	0.004999	0.004999	0.004999	0.004999
***K(+)***	***20.04693***	***7.5***	***25***	***15***
Mg(2+)	1.501201	1.501201	1.501201	1.501201
Mn(2+)	0.099989	0.099989	0.099989	0.099989
Mo(2-)	0.001033	0.001033	0.001033	0.001033
***NH***_4_***(+)***	***20.61395***	***2.5***	***25***	***15***
***NO***_3_***(-)***	***39.40666***	***10***	***50***	***30***
Na(+)	3.202475	3.202475	3.202475	3.202475
PO_4_(3-)	1.249219	1.249219	1.249219	1.249219
SO_4_(2-)	1.738084	1.738084	1.738084	1.738084
Zn(2+)	0.036797	0.036797	0.036797	0.036797

The results are the salt/acid/base formulations required to make each treatment solution. All ions other than the three being varied (italicized and bold) are fixed and unvaried, which illustrates an experimental design free of ion confounding.

The software application Design-Expert^® ^7 (Stat-Ease, Inc, Minneapolis, MN) was used for experimental design construction, model evaluation, and all analyses. Detailed descriptions of the statistical methods used to analyze the data can be found in Niedz and Evens [16] and Evens et al. [21]. Briefly, all possible models from the mean to cubic polynomial were calculated with Design Expert^®^. Initial model selection was based on a battery of adequacy tests [15]. Normality and constant variance were determined graphically; a Box-Cox plot was used to choose the correct transformations [22]. Overly influential data points were identified with DFFITS and DFBETAS plots [23]. Adequate precision of the model was determined by comparing the range of the predicted values at the design points (y) to the average variance (V-bar) of the prediction [15]. Potential outlier points were checked with externally studentized "outlier-t" [14,24] and Cook's Distance [25] graphical plots. R^2^, adjusted-R^2 ^(R^2^_adj_), and predicted-R^2 ^(R^2^_pred_), were estimated for each selected model [26]. ANOVA calculations were conducted for fresh and dry weight responses of both tissue types. The chemical equilibrium modeling software MINEQL^+ ^Ver. 4.5 [27] was used to verify the pH of the treatment solutions. All calculations were temperature corrected and assumed open to the atmosphere with a P_CO2 _at sea level of 10^-3.50 ^atm. The software application Euler 3D ver. 3.1 [28] was used to construct Figure 3.

## Authors' contributions

RPN conceived and coordinated the study. RPN and TJE jointly developed the experimental design, analyzed and interpreted the data. RPN drafted the manuscript. RPN and TJE edited and approved the final manuscript.
